# Books Reviewed

**Published:** 1869-06

**Authors:** 


### Books Reviewed.

A Treatise on the Function of Digestion; its disorders and their treatment. By F. W. Pavy, M. D., F. R. S. From the second London edition. Philadelphia: Henry C. Lea, 1869.

This is a most admirable book, and cannot fail to interest every practitioner of medicine. It contains a discussion upon all the general and special symptoms and conditions in indigestion, and also contains valuable instructions as to the best modes of treating it. We cannot withhold quoting a few sentences, since they not only show the character of the work to some extent, at least, but express facts worth knowing:

"'From numerous trials,' says Dr. Beaumont, 'I am persuaded that moderate exercise conduces considerably to healthy and rapid digestion. The discovery was the result of accident, and contrary to preconceived opinions. I account for it in the following way: Gentle exercise increases the circulation of the system, and the *temperature* of the stomach. This increase of temperature is generally about 1½°. . . . . . Severe and fatiguing exercise, on the contrary, retards digestion. Two reasons present themselves for this: the debility which follows hard labor, of which the stomach partakes; and the depressed temperature of the system consequent upon perspiration and evaporation from the surface.'

"The influence that temperature exerts upon the process of digestion has been already alluded to. In the summary drawn from a large number of observations upon the temperature of Alexis St. Martin's stomach in different states as regards emptiness and repletion, and at different seasons of the year and periods of the day, Dr. Beaumont shows that the temperature is higher during digestion than fasting, and in both these states higher during exercise than repose. For example, with the stomach empty and the body in a state of repose, the temperature ranged between 98° and 100¾°, whilst during exercise it ranged between 100° and 102°; with the stomach full, the range of temperature observed was between 99° and 102° during repose, and during exercise between 100½° and 103°.

"The practice now so common in luxurious life of eating ice-puddings at dinner and ices at dessert is not in harmony, however agreeable at the time it may be, with physiological principles. From well-ascertained facts, it may be positively asserted that lowering the temperature of the interior of the stomach whilst digestion is going on cannot fail to interfere with and retard the completion of the process. Drinking copiously of cold fluids must also exert the same effect as partaking of ice-puddings and ices. Upon one occasion, with the thermometer introduced into St. Martin's stomach whilst in an empty state, Dr. Beaumont noticed that the effect of drinking a quarter of a pint of water at a temperature of 55° was to occasion an immediate fall from 99½° to 70°. The temperature stood at this point for a minute and a half to two minutes, and then began very slowly to rise; but it was not until after half an hour had elapsed that it had risen again to 99°.

"Alcohol is destructive of the power of the gastric juice by throwing down its pepsin in an insoluble state. It is easily shown by means of experiment outside the body, that the effect of the presence of a certain amount of alcohol is to prevent the process of digestion from being carried out. Now, the same must hold good with regard to digestion in the interior of the stomach; and thus is accounted for the vomiting that constitutes so common a consequence of a debauch, and also the fact that the food which has been taken is found to be rejected in an undigested state. The "petit verre" that is frequently taken after a heavy dinner, or to follow an indigestible article of food, is taken with the view of its affording assistanee to digestion; and notwithstanding what has been said, it is not incompatible that it should do so. It is quite consistent tbat a small quantity of spirit taken into the stomach may expedite digestion by its stimulating effect upon the glandular follicles, leading to an increased flow of secretion; whilst a larger quantity, by virtue of its chemical action on the secretion produced—by precipitating as it does its active organic principle, should exert exactly the opposite influence on the result."

